# The impact of interchain hydrogen bonding on *β*‐hairpin stability is readily predicted by molecular dynamics simulation

**DOI:** 10.1002/bip.22671

**Published:** 2015-11-26

**Authors:** Stephan Niebling, Emma Danelius, Ulrika Brath, Sebastian Westenhoff, Máté Erdélyi

**Affiliations:** ^1^ Department of Chemistry and Molecular BiologyUniversity of GothenburgGothenburgSweden

**Keywords:** beta‐hairpin, hydrogen bond, molecular dynamics

## Abstract

Peptides are frequently used model systems for protein folding. They are also gaining increased importance as therapeutics. Here, the ability of molecular dynamics (MD) simulation for describing the structure and dynamics of β‐hairpin peptides was investigated, with special attention given to the impact of a single interstrand sidechain to sidechain interaction. The MD trajectories were compared to structural information gained from solution NMR. By assigning frames from restraint‐free MD simulations to an intuitive hydrogen bond on/off pattern, folding ratios and folding pathways were predicted. The computed molecular model successfully reproduces the folding ratios determined by NMR, indicating that MD simulation may be straightforwardly used as a screening tool in β‐hairpin design. © The Authors. Biopolymers Published by Wiley Periodicals, Inc. Biopolymers (Pept Sci) 104: 703–706, 2015.

## INTRODUCTION

The significance of peptides as therapeutics is rapidly growing. Over one hundred peptidic drugs are marketed for the treatment of diseases such as allergy, asthma, diabetes, inflammation, cancer, as well as cardiovascular and infective illnesses,^1^, ^2^ whereas about 140 peptide candidates are currently in clinical and another 500–600 in preclinical development.^3^ Most of them are small, i.e. are composed of 8‐to‐10 amino acids.^1^ The present approval rate of peptide drugs is twice as compared to that of small molecules. Owing to their rapidly growing importance, the understanding and prediction of the behavior of peptides is receiving increased attention.^4^, ^5^ Peptide foldamers, such as *β*‐hairpins, have gained further importance as suitable model systems for early stages of protein folding.^6^

The dynamic nature of peptides makes their structural analysis a remaining challenge. The solid state structure of oligopeptides does not necessarily coincide with their conformation in solution,^7^ and the commonly utilized NMR restraint‐driven structure calculations are likely to yield averaged conformations that might be more misleading than informative.^8^, ^9^, ^10^

Prediction of the structure of small peptides, such as *β*‐hairpins, was recently attempted by molecular dynamics (MD) simulations, with a number of successful examples.^4^, ^11^, ^12^, ^13^ It should be noted, however, that the outcome of these computations is commonly validated by comparison to an X‐ray derived solid state, or to an NMR‐derived averaged conformation, thus neglecting the inherent dynamic nature of oligopeptides that is best described by a conformational ensemble. Overall, the capability of MD to reproduce dynamic solution conformational ensembles has not yet been as convincingly demonstrated as its ability to refine proteins' structure.

**Figure 1:**
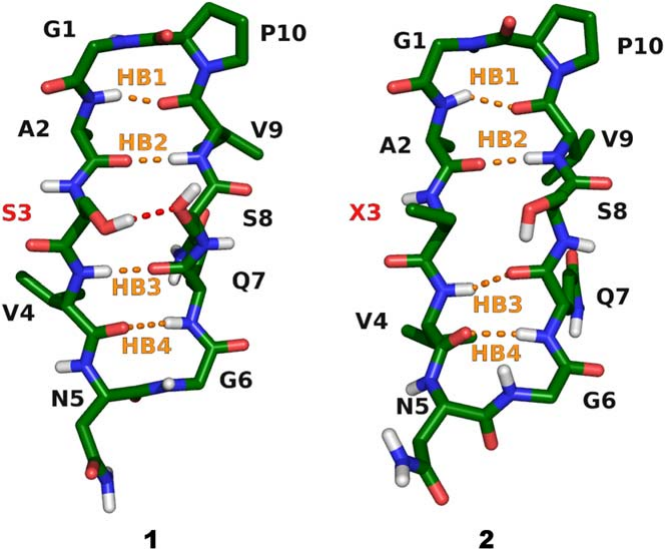
The sequence of cyclic *β*‐hairpins **1** and **2** is identical apart from an OH to CH_3_ substitution at the sidechain of the amino acid at position 3. Whereas the folded *β*‐hairpin conformation of **1** is stabilized by interstrand sidechain to sidechain hydrogen bond of S3 to S8, this interaction is prevented for **2** due to substitution of Ser‐3 to aminobutyric acid (X3). The peptide backbones are shown with carbons in green, nitrogens in blue, oxygens in red, and hydrogens in white. Aliphatic hydrogens are omitted for clarity. Hydrogen bonds are abbreviated as HB1: O_V9_–H_A2_, HB2: O_A2_–H_V9_, HB3: O_Q7_–H_V4_, and HB4: O_V4_–H_Q7_ corresponding to the classification used in Table 1.

Here, the ability of a conventional molecular dynamic simulation protocol to reproduce the dynamic conformational ensemble of a pair of closely related *β*‐hairpins (Figure 1) was examined. This model system allowed evaluation of the ability of the computational technique to describe the influence of a specific interstrand sidechain to sidechain interaction on *β*‐hairpin folding. Our computational output was validated against the solution ensemble that was deduced by NAMFIS (NMR Analysis of Molecular Flexibility in Solution)^8^ from the peptides' solution NMR data.^14^

**Table 1:** The Probabilities of Conformations, their Individual and Averaged Hydrogen Bond Distances (HB1‐4), and the Classification of the Overall Folding of the Most Prevalent Conformations of 1 and 2

		Average Distances/Å					
H‐Bonds	(%)	HB1	HB2	HB3	HB4	Average	Folded?
Peptide **1**							
*oooo*	7	5.87	9.67	8.06	5.00	7.15	u
*oooc*	3	4.14	5.56	4.40	2.30	4.10	u
*cooc*	22	2.29	3.77	3.82	2.18	3.01	u
*cocc*	47	2.40	3.70	2.17	2.13	2.60	f
*ccoc*	4	2.21	2.50	3.70	2.19	2.65	f
*cccc*	9	2.25	2.42	2.18	2.16	2.25	f
Peptide **2**							
*oooo*	14	5.92	8.87	6.95	4.09	6.46	u
*oooc*	7	5.31	7.60	5.14	2.45	5.13	u
*cooc*	28	2.26	3.72	3.83	2.19	3.00	u
*cocc*	29	2.37	3.59	2.26	2.13	2.59	f
*ccoc*	6	2.17	2.49	3.74	2.23	2.66	f
*cccc*	8	2.23	2.37	2.24	2.18	2.25	f

Hydrogen bonds (Figure 1) are characterized as open (*o*) or closed (*c*) corresponding to the cutoff threshold 3 Å. Peptide **1** is predicted to possess 66%, whereas **2** 43% folded *β*‐hairpin conformation, which compare well to the experimentally determined 88% and 55%, respectively.^14^ Here u = unfolded, f = folded. The full tables with all the theoretically possible 16 population groups is shown in the Supporting Information (Table SI).

## METHODS

MD simulations were performed with GROMACS 4.5.5^15^, ^16^ using the OPLS‐AA force field,^17^, ^18^ with parameters for the non‐natural amino acid ABU derived from parameters of leucine C_*β*_ and isoleucine C_*γ*_. Parameters for the solvent dimethylsulfoxide (DMSO) were used as implemented in Gromacs 4.5.5. Initial coordinates for **1** and **2** (Figure 1) were taken from an output structure of the NAMFIS analysis of their solution NMR data.^14^

The peptides were solvated in a cubic box with periodic boundary conditions and a side length of ∼36 Å (10 Å initial minimum distance of solute to all boundaries) comprising the peptide and ∼400 DMSO molecules. For both peptides, the same molecular dynamics protocol was used. After a steepest descent energy minimization (convergence criteria 500,000 steps or maximum force *<*10 kJ mol^*−*1^ nm^*−*1^) two 100 ps equilibration MD runs were performed. The first one in the constant particle number, volume, temperature ensemble (NVT; with modified Berendsen thermostat with velocity rescaling^19^ at 300 K and a 0.1 ps timestep; separate heat baths for peptide and solvent); the second one in the constant particle number, pressure, temperature ensemble (NPT; Parrinello–Rahman pressure coupling^20^, ^21^ at 1 bar with a compressibility of 4.5 × 10^*−*5^ bar^*−*1^ and a 2 ps time constant). During both equilibration runs, a position restraint potential with a force constant of 1000 kJ mol^*−*1^ nm^*−*2^ was added to all peptide atoms. To generate coordinates and velocities for the following production runs, a 100 ps simulation with position restraints (same as for the equilibration runs) was used. Coordinates and velocities at every 10 ps were used for the production runs (11 × 400 ns) which resulted in a total simulation time of 4.4 µs for each peptide. For all MD simulations the leap‐frog integrator was used with a time step of 2 fs. Coordinates were saved every 2 ps. The same temperature and pressure coupling schemes as applied for the equilibration runs were used for the subsequent MD simulations. All bonds to hydrogen atoms were constrained using the linear constrained solver (LINCS)^22^ with an order of 4 and one iteration. A grid‐based neighbor list with a threshold of 10 Å was used and updated every five steps (10 fs). The particle‐mesh Ewald method^23^, ^24^ was used for long‐range electrostatic interactions above 10 Å with a fourth order interpolation and a maximum spacing for the FFT grid of 1.6 Å. Lennard–Jones interactions were cutoff above 10 Å. A long range dispersion correction for energy and pressure was used to compensate for the Lennard–Jones interaction cutoff.^16^

## RESULTS AND DISCUSSION

### Population Analysis

MD frames were classified according to the presence or absence of the possible interchain hydrogen bonds of the peptides' folded *β*‐hairpin conformation, using the on/off scheme for HB1‐4 shown in Figure 1. Hydrogen bonds were detected with the software MDAnalysis^25^ with a distance threshold of 3 Å and an angle lower limit of 120 °. If these criteria were met, a hydrogen bond was labelled as *c* (closed), otherwise it was labelled as *o* (opened). The four possible hydrogen bonds yield 2^4^ = 16 theoretically available hydrogen bond patterns, yet only around half of the combinations were observed to be significantly populated. The contribution of the six most abundant hydrogen bond patterns is shown in Table 1, whereas the populations of all conformations are shown in Supporting Information Table SI.

Overall folding of **1** and **2** was analysed by determining the average distances of the interchain hydrogen bonds HB1‐4 (Figure 1) and assigning the structures that possess an average HB distance below the 3 Å threshold as folded, whilst those with an average distance above this threshold as unfolded (last column in Table 1). This characterization of the MD trajectory yields an overall folding of 66% for **1** and 43% for **2**, which are in agreement with the experimentally derived 88% versus 55%, respectively, within the limitations of the applied techniques.^14^

The hydrogen bond distances of the folded conformations *cccc, ccoc*, and *cocc* of **1** and **2**, shown in Table 1, were comparable. This confirmed that the OH to CH_3_ substitution at amino acid 3 does not lead to any distortion of the overall backbone conformation of the peptide. Interestingly, barely 3% of the frames in the trajectory of **1** display the S3–S8 interstrand hydrogen bond that may not explain the higher β‐hairpin propensity of **1** as compared to **2**. The S3 sidechain of **1**, however, can additionally form intrastrand hydrogen bond with the carbonyl oxygen of S3 (18%) and that of A2 (10%), which may stabilize an extended β‐strand and thereby the β‐hairpin conformation (for additional details see Supporting Information Table SII). These hydrogen bonds are predicted to preferentially occur in combination with the *cocc* hydrogen bond pattern (Supporting Information Table SIII), explaining its higher prevalence for **1** (47%) as compared to **2** (29 %), and possibly the higher overall folding rate.

### Population Change Analysis

**Figure 2:**
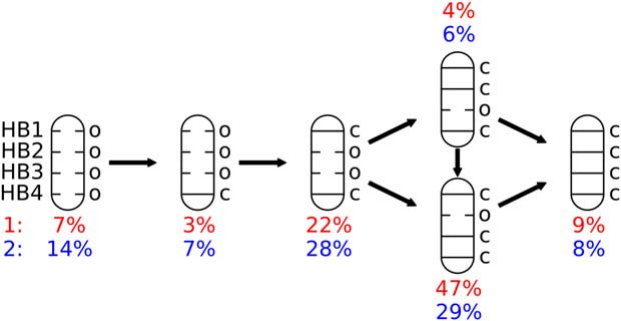
Folding pathway of peptides **1** and **2**. The most probable folding route from the completely unfolded *oooo* state to the completely folded *cccc* conformation was derived from the population change maps shown in Table 2. The probability of each state at room temperature is denoted below the hydrogen bond schemes. Probabilities of the less populated states are given in the Supporting Information.

Molecular dynamics trajectories may provide a stochastic model for biological processes, and were previously used to characterize folding and molecular recognition events.^26^, ^27^, ^28^, ^29^ Population change maps, shown in Table 2, were generated by counting the number of transitions, in percentage, in the MD trajectory between the hydrogen bond patterns shown in Table 1. The line in Table 2 specifies from which population the transition starts and the column in which population it ends. The population analysis of **1** and **2** reveals that a majority of transitions return to the same population group they started from, as indicated by the highest probabilities belonging to the diagonals. The most probable folding pathway is indicated by the highest values in respective rows of Table 2, ignoring the diagonal value, and is shown in Figure 2.

**Table 2:** Population Change Maps for the Conformations of Peptides **1** and **2**

Peptide **1**						
	To					
From	*oooo*	*oooc*	*cooc*	*cocc*	*ccoc*	*cccc*
*oooo*	94%	4%	0%	0%	0%	0%
*oooc*	9%	49%	23%	8%	3%	1%
*cooc*	0%	3%	72%	14%	7%	2%
*cocc*	0%	1%	6%	79%	1%	8%
*ccoc*	0%	2%	39%	8%	37%	9%
*cccc*	0%	0%	6%	39%	3%	46%
Peptide **2**						
	To					
From	*oooo*	*oooc*	*cooc*	*cocc*	*ccoc*	*cccc*
*oooo*	87%	10%	0%	0%	0%	0%
*oooc*	21%	65%	8%	2%	1%	0%
*cooc*	0%	2%	75%	11%	8%	2%
*cocc*	0%	1%	11%	73%	1%	9%
*ccoc*	0%	1%	39%	6%	40%	7%
*cccc*	0%	0%	8%	34%	5%	47%

Classification and quantification of these groups are given in Table 1. Population change maps for all the theoretically possible 16 population groups are shown in the Supporting Information (Table SIV).

Both peptides follow the same order of hydrogen bond formation. Thus, starting from the fully unfolded state, HB4 is formed first yielding structure *oooc*. This is followed by formation of HB1 that leads to the structure *cooc*. Next, either HB2 or HB3 is closed with somewhat different probabilities, giving first *cocc* or *ccoc* and then leading to the fully folded *β*‐hairpin, *cccc*. The sequence of hydrogen bond formation upon folding may offer valuable information for designing peptides with specific structural properties. It should be noted that folding pathways may be sensitive towards force field parameters,^30^ and the above proposed folding pathway should therefore be interpreted with care.

## SUMMARY

The ability of a simple, standard MD simulation protocol for prediction of *β*‐hairpin folding was demonstrated. A straightforward hydrogen bond analysis of the frames in an MD trajectory was shown to yield folding ratios that are in good agreement with experimental (NMR) observations.^14^ Moreover, we have shown that the applied MD simulation can predict the importance of a specific secondary interaction, here S3–S8, on folding ratios. It may also provide an improved understanding of the impact of amide to amide interstrand hydrogen bonds and the dynamics of peptide folding.

## Supporting information

Additional Supporting Information may be found in the online version of this article

Supporting Information
