# Supplementary material for: The impact of interchain hydrogen bonding on β‐hairpin stability is readily predicted by molecular dynamics simulation
Source: Biopolymers. 2015 Nov 26;104(6):703–6. doi: 10.1002/bip.22671 (PMC4744756; doi:10.1002/bip.22671)
Supplement: Supplementary file 1 — Supporting Information [file BIP-104-703-s001.docx]

**Electronic Supplementary Information**

Table S1: Full list of conformer groups

Table S2: Hydrogen bond occurrence in trajectories

Table S3: Side chain hydrogen bonds of S3 for peptide **1**

Table S4: Full population change maps

Table S1: Conformations with the possible variations of intramolecular hydrogen bond patterns for **1** and **2**, and the corresponding average distances (for abbreviations see Fig 1). The first column contains a classification of the hydrogen bonds HB1–4 (cf. fig 1 in open (*o*) and closed (*c*)). In the last column the average distance of all four hydrogen bonds is used to classify the whole structure as folded (*f* ) or unfolded (*u* ), in order to determine the overall folding ratio. For both classifications, a distance threshold of 3 Å was used.

Peptide **1**: ∑f = 66% (Lit.^14^ 88 %)

|  |  | Average distances / Å | | | | |  |
| --- | --- | --- | --- | --- | --- | --- | --- |
| H-bonds | % | HB1 | HB2 | HB3 | HB4 | Average | Folded? |
| oooo | 7 | 5.87 | 9.67 | 8.06 | 5.00 | 7.15 | u |
| oooc | 3 | 4.14 | 5.56 | 4.40 | 2.30 | 4.10 | u |
| cooc | 22 | 2.29 | 3.77 | 3.82 | 2.18 | 3.01 | u |
| cocc | 47 | 2.40 | 3.70 | 2.17 | 2.13 | 2.60 | f |
| ccoc | 4 | 2.21 | 2.50 | 3.70 | 2.19 | 2.65 | f |
| cccc | 9 | 2.25 | 2.42 | 2.18 | 2.16 | 2.25 | f |
| oocc | 5 | 3.22 | 4.08 | 2.20 | 2.13 | 2.91 | f |
| cooo | 1 | 2.41 | 4.83 | 5.10 | 4.25 | 4.15 | u |
| ccco | 1 | 2.19 | 2.05 | 2.10 | 3.74 | 2.52 | f |
| ccoo | 1 | 2.16 | 2.38 | 4.05 | 3.53 | 3.03 | u |
| coco | 0 | 2.40 | 3.85 | 2.30 | 3.31 | 2.96 | f |
| occc | 0 | 2.94 | 2.52 | 2.17 | 2.17 | 2.45 | f |
| ococ | 0 | 2.93 | 2.62 | 3.65 | 2.17 | 2.84 | f |
| ooco | 0 | 3.28 | 4.12 | 2.31 | 3.37 | 3.27 | u |
| occo | 0 | 2.91 | 2.17 | 2.08 | 3.74 | 2.72 | f |
| ocoo | 0 | 2.91 | 2.53 | 4.00 | 3.49 | 3.23 | u |

Peptide **2**: ∑f = 43% (Lit.^14^ 55 %)

|  |  | Average distances / Å | | | | |  |
| --- | --- | --- | --- | --- | --- | --- | --- |
| H-bonds | % | HB1 | HB2 | HB3 | HB4 | Average | Folded? |
| oooo | 14 | 5.92 | 8.87 | 6.95 | 4.09 | 6.46 | u |
| oooc | 7 | 5.31 | 7.60 | 5.14 | 2.45 | 5.13 | u |
| cooc | 28 | 2.26 | 3.72 | 3.83 | 2.19 | 3.00 | u |
| cocc | 29 | 2.37 | 3.59 | 2.26 | 2.13 | 2.59 | f |
| ccoc | 6 | 2.17 | 2.49 | 3.74 | 2.23 | 2.66 | f |
| cccc | 8 | 2.23 | 2.37 | 2.24 | 2.18 | 2.25 | f |
| oocc | 4 | 3.77 | 4.87 | 2.25 | 2.14 | 3.25 | u |
| cooo | 3 | 2.39 | 4.46 | 5.07 | 4.38 | 4.08 | u |
| ccoo | 1 | 2.15 | 2.38 | 4.19 | 3.63 | 3.09 | u |
| ccco | 0 | 2.20 | 2.04 | 2.25 | 3.29 | 2.45 | f |
| occc | 0 | 2.93 | 2.42 | 2.18 | 2.20 | 2.43 | f |
| coco | 0 | 2.39 | 3.97 | 2.40 | 3.19 | 2.99 | f |
| ococ | 0 | 2.91 | 2.60 | 3.68 | 2.22 | 2.85 | f |
| ooco | 0 | 4.00 | 5.27 | 2.29 | 3.16 | 3.68 | u |
| ocoo | 0 | 2.85 | 2.55 | 4.22 | 3.82 | 3.36 | u |
| occo | 0 | 2.94 | 2.15 | 2.23 | 3.23 | 2.64 | f |

Table S2: Hydrogen bond occurrence in the trajectories of **1** and **2**. For hydrogen bond detection the python module MDAnalysis^25^ was used with a distance threshold of 3 Å and a minimum angle of 120°. Only hydrogen bonds with a probability higher than 1% are listed.

| Peptide **1** | |  |  | |  |  | Peptide **2** | |  | | | |
| --- | --- | --- | --- | --- | --- | --- | --- | --- | --- | --- | --- | --- |
| Donor | |  | Acceptor | |  |  | Donor | |  | Acceptor | |  |
| Residue | Atom |  | Residue | Atom | % |  | Residue | Atom |  | Residue | Atom | % |
|  |  |  |  |  |  |  | G1 | H |  | S8 | O | 3 |
|  |  |  |  |  |  |  | G1 | H |  | V9 | O | 3 |
| A2 | H |  | V9 | O | 85 |  | A2 | H |  | V9 | O | 76 |
| A2 | H |  | P10 | O | 6 |  | A2 | H |  | P10 | O | 4 |
| S3 | HG |  | G1 | O | 2 |  |  |  |  |  |  |  |
| S3 | HG |  | A2 | O | 10 |  |  |  |  |  |  |  |
| S3 | HG |  | S3 | O | 18 |  |  |  |  |  |  |  |
| S3 | H |  | Q7 | OE1 | 1 |  |  |  |  |  |  |  |
| S3 | HG |  | S8 | OG | 3 |  |  |  |  |  |  |  |
| V4 | H |  | A2 | O | 2 |  | V4 | H |  | A2 | O | 6 |
| V4 | H |  | Q7 | O | 63 |  | V4 | H |  | Q7 | O | 41 |
| V4 | H |  | Q7 | OE1 | 1 |  | V4 | H |  | Q7 | OE1 | 8 |
| V4 | H |  | S8 | OG | 5 |  | V4 | H |  | S8 | OG | 5 |
| G6 | H |  | V4 | O | 3 |  | G6 | H |  | V4 | O | 3 |
|  |  |  |  |  |  |  | Q7 | HE22 |  | G1 | O | 2 |
| Q7 | H |  | V4 | O | 91 |  | Q7 | H |  | V4 | O | 82 |
|  |  |  |  |  |  |  | Q7 | H |  | N5 | O | 2 |
| Q7 | HE21 |  | G6 | O | 64 |  | Q7 | HE21 |  | G6 | O | 53 |
| Q7 | HE21 |  | V9 | O | 2 |  | Q7 | HE21 |  | V9 | O | 6 |
| S8 | HG |  | A2 | O | 3 |  | S8 | HG |  | A2 | O | 4 |
| S8 | HG |  | V4 | O | 48 |  | S8 | HG |  | V4 | O | 46 |
| S8 | H |  | Q7 | OE1 | 1 |  | S8 | H |  | Q7 | OE1 | 3 |
| S8 | HG |  | Q7 | O | 6 |  | S8 | HG |  | Q7 | O | 4 |
| S8 | HG |  | S8 | O | 5 |  | S8 | HG |  | S8 | O | 5 |
| V9 | H |  | A2 | O | 15 |  | V9 | H |  | A2 | O | 15 |
| V9 | H |  | Q7 | O | 30 |  | V9 | H |  | Q7 | O | 35 |
|  |  |  |  |  |  |  | V9 | H |  | Q7 | OE1 | 3 |

Table S3: Three most common hydrogen bonds of the S3 side chain hydroxyl group (S3_HG) of **1** with nearby hydrogen bond acceptors: the backbone carbonyl group of A2 (A2_O; in 18% of all frames), the backbone carbonyl group of S3 (S3_O; in 10% of all frames), and the side chain hydroxyl group of S8 (S8_OG; in 3% of all frames, cf. table S2). The hydrogen bonds to the backbone carbonyls of A2 and S3 mainly appear in frames assigned to the hydrogen bond pattern *cocc*. This leads to a higher percentage of the latter hydrogen bond pattern for **1** compared to **2** that might explain the β-hairpin stabilizing effect of the S3 side chain. The side chain to side chain hydrogen bond between S3 and S8 occurs with similar percentages for the hydrogen bond pattern *cocc* and *cccc*.

|  | All frames | |  | Frames with hydrogen bonds | | | | | | | |
| --- | --- | --- | --- | --- | --- | --- | --- | --- | --- | --- | --- |
|  |  | |  | S3_HG – S3_O | |  | S3_HG – A2_O | |  | S3_HG – S8_OG | |
| HB Pattern | Total | Rel. |  | Total | Rel. |  | Total | Rel. |  | Total | Rel. |
| *oooo* | 153403 | 7% |  | 4012 | 1% |  | 9315 | 5% |  | 242 | 0% |
| *oooc* | 62647 | 3% |  | 3422 | 1% |  | 1163 | 1% |  | 1145 | 2% |
| *cooc* | 461652 | 22% |  | 65453 | 17% |  | 26504 | 13% |  | 3549 | 7% |
| *cocc* | 1002459 | 47% |  | 242548 | 63% |  | 143111 | 70% |  | 19079 | 35% |
| *ccoc* | 79439 | 4% |  | 9756 | 3% |  | 2291 | 1% |  | 4311 | 8% |
| *cccc* | 197549 | 9% |  | 33704 | 9% |  | 11844 | 6% |  | 17487 | 32% |
| *oocc* | 104910 | 5% |  | 21128 | 5% |  | 9117 | 4% |  | 3892 | 7% |
| *cooo* | 25483 | 1% |  | 1549 | 0% |  | 338 | 0% |  | 272 | 1% |
| *ccco* | 16385 | 1% |  | 803 | 0% |  | 59 | 0% |  | 1219 | 2% |
| *ccoo* | 13641 | 1% |  | 588 | 0% |  | 20 | 0% |  | 1685 | 3% |
| *coco* | 5711 | 0% |  | 992 | 0% |  | 574 | 0% |  | 305 | 1% |
| *occc* | 5482 | 0% |  | 624 | 0% |  | 276 | 0% |  | 732 | 1% |
| *ococ* | 2220 | 0% |  | 162 | 0% |  | 33 | 0% |  | 200 | 0% |
| *ooco* | 745 | 0% |  | 109 | 0% |  | 32 | 0% |  | 108 | 0% |
| *occo* | 381 | 0% |  | 7 | 0% |  | 1 | 0% |  | 37 | 0% |
| *ocoo* | 260 | 0% |  | 10 | 0% |  | 0 | 0% |  | 50 | 0% |
| Sum | 2132367 |  |  | 384867 (18%) | |  | 204678 (10%) | |  | 54313 (3%) | |
|  |  |  |  |  |  |  |  |  |  |  |  |

Table S4: Full population change maps for the seven most populated groups of peptides **1** and **2**.

| to  from |  |  |  |  |  |  |  |  |  |  |  |  |  |  |  |  |
| --- | --- | --- | --- | --- | --- | --- | --- | --- | --- | --- | --- | --- | --- | --- | --- | --- |
|  | *oooo* | *oooc* | *cooc* | *cocc* | *ccoc* | *cccc* | *oocc* | *cooo* | *ccoo* | *ccco* | *occc* | *coco* | *ococ* | *ooco* | *ocoo* | *occo* |
| *oooo* | 94% | 4% | 0% | 0% | 0% | 0% | 0% | 2% | 0% | 0% | 0% | 0% | 0% | 0% | 0% | 0% |
| *oooc* | 9% | 49% | 23% | 8% | 3% | 1% | 5% | 1% | 0% | 0% | 0% | 0% | 0% | 0% | 0% | 0% |
| *cooc* | 0% | 3% | 72% | 14% | 7% | 2% | 1% | 1% | 0% | 0% | 0% | 0% | 0% | 0% | 0% | 0% |
| *cocc* | 0% | 1% | 6% | 79% | 1% | 8% | 6% | 0% | 0% | 0% | 0% | 0% | 0% | 0% | 0% | 0% |
| *ccoc* | 0% | 2% | 39% | 8% | 37% | 9% | 1% | 1% | 0% | 3% | 0% | 0% | 1% | 0% | 0% | 0% |
| *cccc* | 0% | 0% | 6% | 39% | 3% | 46% | 2% | 0% | 2% | 0% | 0% | 1% | 0% | 0% | 0% | 0% |
| *oocc* | 0% | 3% | 4% | 56% | 0% | 5% | 31% | 0% | 0% | 0% | 0% | 1% | 0% | 0% | 0% | 0% |
| *cooo* | 10% | 1% | 18% | 2% | 2% | 0% | 0% | 58% | 0% | 7% | 0% | 0% | 0% | 0% | 0% | 0% |
| *ccco* | 0% | 0% | 0% | 2% | 1% | 19% | 0% | 0% | 71% | 3% | 2% | 0% | 0% | 0% | 1% | 0% |
| *ccoo* | 1% | 0% | 4% | 0% | 17% | 3% | 0% | 13% | 3% | 57% | 0% | 0% | 0% | 0% | 0% | 1% |
| *coco* | 0% | 0% | 3% | 61% | 0% | 6% | 5% | 2% | 5% | 0% | 15% | 0% | 0% | 2% | 0% | 0% |
| *occc* | 0% | 2% | 4% | 31% | 3% | 42% | 10% | 0% | 2% | 0% | 0% | 5% | 1% | 0% | 0% | 0% |
| *ococ* | 0% | 12% | 27% | 6% | 33% | 8% | 3% | 0% | 0% | 2% | 0% | 1% | 6% | 0% | 0% | 0% |
| *ooco* | 2% | 2% | 1% | 37% | 0% | 5% | 26% | 1% | 4% | 1% | 11% | 1% | 0% | 9% | 1% | 0% |
| *occo* | 0% | 0% | 1% | 3% | 1% | 18% | 2% | 0% | 65% | 1% | 2% | 3% | 0% | 2% | 4% | 0% |
| *ocoo* | 4% | 1% | 5% | 0% | 17% | 5% | 0% | 14% | 5% | 43% | 0% | 0% | 2% | 0% | 1% | 4% |
|  |  |  |  |  |  |  |  |  |  |  |  |  |  |  |  |  |
| to  from |  |  |  |  |  |  |  |  |  |  |  |  |  |  |  |  |
|  | *oooo* | *oooc* | *cooc* | *cocc* | *ccoc* | *cccc* | *oocc* | *cooo* | *ccoo* | *ccco* | *occc* | *coco* | *ococ* | *ooco* | *ocoo* | *occo* |
| *oooo* | 87% | 10% | 0% | 0% | 0% | 0% | 0% | 2% | 0% | 0% | 0% | 0% | 0% | 0% | 0% | 0% |
| *oooc* | 21% | 65% | 8% | 2% | 1% | 0% | 2% | 1% | 0% | 0% | 0% | 0% | 0% | 0% | 0% | 0% |
| *cooc* | 0% | 2% | 75% | 11% | 8% | 2% | 0% | 1% | 0% | 0% | 0% | 0% | 0% | 0% | 0% | 0% |
| *cocc* | 0% | 1% | 11% | 73% | 1% | 9% | 5% | 0% | 0% | 0% | 0% | 0% | 0% | 0% | 0% | 0% |
| *ccoc* | 0% | 1% | 39% | 6% | 40% | 7% | 0% | 1% | 5% | 0% | 0% | 0% | 0% | 0% | 0% | 0% |
| *cccc* | 0% | 0% | 8% | 34% | 5% | 47% | 2% | 0% | 0% | 2% | 1% | 0% | 0% | 0% | 0% | 0% |
| *oocc* | 0% | 3% | 3% | 37% | 0% | 3% | 52% | 0% | 0% | 0% | 0% | 0% | 0% | 0% | 0% | 0% |
| *cooo* | 8% | 1% | 13% | 1% | 2% | 0% | 0% | 66% | 8% | 0% | 0% | 0% | 0% | 0% | 0% | 0% |
| *ccoo* | 1% | 0% | 4% | 0% | 19% | 2% | 0% | 17% | 55% | 1% | 0% | 0% | 0% | 0% | 1% | 0% |
| *ccco* | 0% | 0% | 0% | 3% | 3% | 56% | 0% | 0% | 8% | 27% | 1% | 1% | 0% | 0% | 0% | 0% |
| *occc* | 0% | 1% | 5% | 27% | 3% | 47% | 8% | 0% | 0% | 2% | 6% | 0% | 0% | 0% | 0% | 0% |
| *coco* | 0% | 1% | 6% | 67% | 0% | 6% | 5% | 4% | 0% | 1% | 0% | 8% | 0% | 1% | 0% | 0% |
| *ococ* | 1% | 9% | 31% | 6% | 37% | 6% | 2% | 1% | 4% | 0% | 0% | 0% | 3% | 0% | 0% | 0% |
| *ooco* | 1% | 3% | 1% | 23% | 0% | 2% | 59% | 1% | 0% | 1% | 0% | 3% | 0% | 5% | 0% | 0% |
| *ocoo* | 8% | 0% | 5% | 0% | 14% | 0% | 0% | 21% | 48% | 2% | 0% | 0% | 0% | 0% | 2% | 0% |
| *occo* | 0% | 0% | 0% | 6% | 3% | 54% | 0% | 1% | 7% | 21% | 5% | 0% | 0% | 0% | 0% | 4% |

Peptide **1**

15

Peptide **2**
